# The mechanical cell – the role of force dependencies in synchronising protein interaction networks

**DOI:** 10.1242/jcs.259769

**Published:** 2022-11-18

**Authors:** Benjamin T. Goult, Magdaléna von Essen, Vesa P. Hytönen

**Affiliations:** ^1^ School of Biosciences, University of Kent, Canterbury CT2 7NJ, Kent, UK; ^2^ Faculty of Medicine and Health Technology, Tampere University, FI-33100 Tampere, Finland; ^3^ Fimlab Laboratories, FI-33520 Tampere, Finland

**Keywords:** Mechanotransduction, Mechanosensing, Motor proteins, Cytoskeleton, Disease-associated mutations, Talin, Force-dependent switches

## Abstract

The role of mechanical signals in the proper functioning of organisms is increasingly recognised, and every cell senses physical forces and responds to them. These forces are generated both from outside the cell or via the sophisticated force-generation machinery of the cell, the cytoskeleton. All regions of the cell are connected via mechanical linkages, enabling the whole cell to function as a mechanical system. In this Review, we define some of the key concepts of how this machinery functions, highlighting the critical requirement for mechanosensory proteins, and conceptualise the coupling of mechanical linkages to mechanochemical switches that enables forces to be converted into biological signals. These mechanical couplings provide a mechanism for how mechanical crosstalk might coordinate the entire cell, its neighbours, extending into whole collections of cells, in tissues and in organs, and ultimately in the coordination and operation of entire organisms. Consequently, many diseases manifest through defects in this machinery, which we map onto schematics of the mechanical linkages within a cell. This mapping approach paves the way for the identification of additional linkages between mechanosignalling pathways and so might identify treatments for diseases, where mechanical connections are affected by mutations or where individual force-regulated components are defective.

## Teaser

**Summary:** We define the cell as mechanical machinery, highlighting the role of mechanosensory proteins and conceptualising the coupling of mechanical linkages to mechanochemical switches that enables conversion of forces into biological signals.

## Introduction

Every living cell receives physical signals from its environment. These physical signals, such as forces generated by motor proteins, mechanical load, shearing forces, flow and pressure are transduced into biological signals by complex mechanosensitive machinery in a process known as mechanotransduction. Much of this response to physical cues is achieved by a sensory network of receptor complexes that form at contact points between the cell and its surroundings as proposed by Ingber almost 30 years ago (Ingber, 1997, 2003; Wang et al., 2009). However, there are also many mechanoreceptors inside the cell that respond to changes in the tension of the cytoskeleton of the cell (Gautel, 2011; Goult et al., 2018; Margadant et al., 2011; Sawada et al., 2006; Wang et al., 2019a), nuclear shape (Earle et al., 2020; Nava et al., 2020; Stephens et al., 2019) and the behaviour of organelles (Helle et al., 2017).

Pioneering work from the Sheetz laboratory has demonstrated that the cytoskeleton responds to mechanical load, identifying p130Cas (also known as BCAR1) as a force-responsive protein (Sawada and Sheetz, 2002). Closer inspection revealed that phosphorylation of p130Cas is modulated by mechanical tension (Sawada et al., 2006). Since the discovery of p130Cas as a mechanoregulated protein, the field of mechanobiology has exploded and now hundreds of proteins that are mechanoregulated and integrated into complex mechanical linkages have been identified. The large number of force-responsive proteins in cells is exemplified by studies that quantify proteins present at cell–extracellular matrix (ECM) adhesions when the motor protein, myosin II is inhibited (Kuo et al., 2011; Schiller et al., 2011); 459 of the 905 proteins identified in these complexes change in abundance when mechanical load is reduced (Kuo et al., 2011; Schiller et al., 2011).

**Fig. 1.**
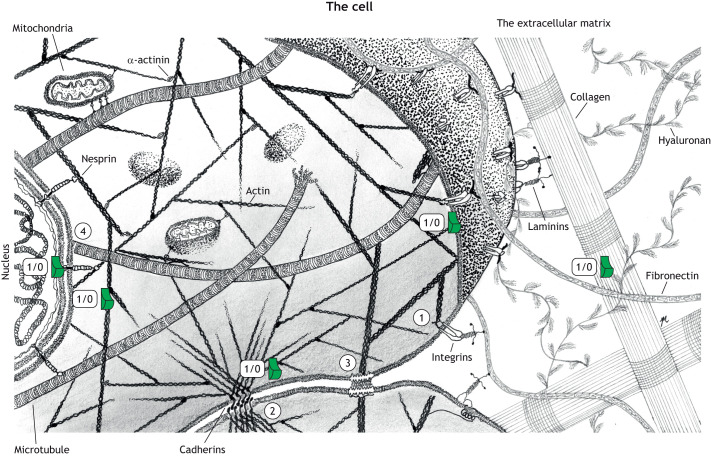
**The complex mechanical linkages that scaffold and form the cells machinery.** An artistic illustration of a cell connected to a neighbouring cell (bottom) and tightly connected to the extracellular matrix (light grey; right). The matrix contains fibrous molecules, such as collagen and fibronectin, and also flexible molecules, such as hyaluronan. On the intracellular side, cellular components are tightly interlinked, and the mechanical connections formed by the cytoskeleton connect the cell membrane, cellular organelles and the nucleus. (1–3) Three of the mechanosensory complexes that connect the exterior of the cell to the cytoskeleton. (1) the integrin-mediated focal adhesions, (2) the cadherin-mediated adherens junctions and (3) the desmosome. Two of the three major cytoskeletal systems, actin and microtubules are shown and these form mechanical linkages that couple complexes 1–3 to each other and to cellular organelles, such as the mitochondria, and via the LINC complex to the nucleus (4). Mechanosensitive proteins localised at the ends of these linkages provide an array of binary switches, indicated by a ‘1/0’ and a green ‘light switch’, that can be operated by the force-generation machinery of the cell. The mechanical coupling of disparate parts of the cell enables long-range communication both within and between cells and so we present the idea of the cell as a complex array of interconnected, mechanically-operated switches functioning as a machine. Illustration generated by Iiris Mustonen, Tampere University, Finland.

The concept that mechanical signals are transmitted to the nucleus leading to changes in gene expression is well established (Cooper and Giancotti, 2019; Elosegui-Artola et al., 2018; Engler et al., 2006; Sun et al., 2016; Wang et al., 2009), and here we introduce how mechanical forces are sensed by proteins, acting as mechanical switches, and how networks of mechanical switches, connected by mechanical linkages, couple all regions of the cell (Fig. 1). The attachments of cells to each other and to the ECM are critical for normal tissue development as large mechanosensitive signalling complexes form at these attachment sites, which enable cells to sense the physical properties of the ECM to guide cell behaviour and differentiation, and to transmit physical signals into its environment (Hytönen and Wehrle-Haller, 2014). Furthermore, the mechanical linkages that emanate from these attachments are fundamental to multicellular life as they connect to other cellular compartments, integrating mechanical cues into biochemical responses that control cellular functioning.

Great complexity emerges from the coupling of the force generation and force-sensing machinery, and many force dependencies arise in the protein interaction networks. The aim of this Review is to present a global view of the cell where these assemblies working together in synchrony represent a vast mechanosensitive network of mechanical switches, connected via mechanical linkages functioning as a dynamic, complex machine that coordinates cell shape, form and function (Fig. 1). Many genetic diseases occur as a result of defective components of these mechanosensitive structures, and in the second part we consider how these defects map onto the mechanical linkages. By viewing these machineries as a whole ‘mechanical cell’, a deeper understanding of the diverse disease states that result from mutations in these linkages can be appreciated which might help translation into novel clinical treatments.

## Molecular mechanisms in mechanosignalling

Mechanosignalling involves multiple components that traverse the different levels of organisation in a cell and across the entire organism. As a result, mechanoregulated channels (Martinac, 2012; Yao et al., 2020 preprint), membrane dynamics (Butler et al., 2001; Haidekker et al., 2000; reviewed in Yamamoto and Ando, 2018), conformational changes in proteins (del Rio et al., 2009; Johnson et al., 2007; Matsui et al., 2012; Yao et al., 2016) and resultant regulation of gene expression (Chien et al., 1998) have all been shown to contribute to mechanosignalling. In this Review, we mainly focus on the regulation of protein conformation, as it represents a ubiquitous mechanism for cellular mechanosensing.

### Protein conformation – plenty of conformational space to explore

Proteins adopt a limited number of conformations in solution, and the concept that proteins fold into their ‘native state’ is widely accepted (Englander and Mayne, 2014). As a result of enormous efforts, >190,000 protein structures have been determined, with most revealing one major conformer. Knowledge of how proteins adopt a native state has led to the development of software, such as AlphaFold (Senior et al., 2020), that can predict protein conformations from sequence data alone by exploiting the existing structural information.

Nevertheless, a surprisingly high proportion of our proteome (∼30%) is expected to be partially disordered, with the fraction of disordered proteins higher in complex multicellular organisms than in simple unicellular organisms (Mészáros et al., 2009). Locally disordered regions within proteins are biologically relevant and are enriched in sequences that mediate cellular signalling functions (Wright and Dyson, 2015). Notably, intrinsically disordered proteins participate in diverse cellular functions, such as phase separation (Martin and Holehouse, 2020), enabling accessibility to ligand-binding sites (Cermakova et al., 2021), defining zones of influence of proteins tethered at one end (Barnett and Goult, 2022 preprint), as well as being enriched in post-translational modifications (PTMs), such as phosphorylation (Khoury et al., 2011).

In this section, we discuss key concepts of mechanosignalling associated with the mechanomodulation of protein conformation. The simplest notion of this can be imagined with a protein domain that adopts a low-energy folded conformation, but upon applied force, undergoes a conformational change, unfolding to either a linear peptide chain or a partially unfolded intermediate state. In an ideal mechanosensor, this force-induced conformational change is reversible to allow dynamic sensing and responding to mechanical force. A textbook example is shown by the interactions of the mechanosensitive proteins talin and vinculin (del Rio et al., 2009; Wang et al., 2021; Yao et al., 2014a, 2016) where mechanical unfolding of talin (herein referring generically to both talin 1 and 2) exposes binding sites for vinculin that are cryptic in the folded conformation.

#### How can mechanical load influence protein conformation?

The effects of applied forces on protein conformation can be studied experimentally. Single-molecule methods, such as atomic force microscopy (AFM) (Puchner and Gaub, 2009), and magnetic (Zhao et al., 2017) and optical (Bustamante et al., 2021) tweezers, as well as steered molecular dynamics (SMD) simulations (Isralewitz et al., 2001), all enable detailed visualisation of these processes. These studies are revealing that mechanical load influences the conformational space of proteins (Hytönen and Vogel, 2008; Pruitt et al., 2014). In the absence of mechanical load, or under low force, a particular protein conformation might be dominant, but once a certain force threshold is exceeded, the protein might switch into a completely unfolded state. These represent the extreme states of the protein, the fully folded and fully unfolded states, and are easy to visualise in solution (as one can denature the protein with temperature, denaturant etc.). However, under limited force, the protein might exist in conformations rarely seen under equilibrium conditions. These are often referred to as intermediate states (Imparato and Pelizzola, 2008; Li et al., 2005; Mykuliak et al., 2018; Schwaiger et al., 2004) and such conformations might only exist under mechanical load where they could even represent the lowest energy conformation (Mykuliak et al., 2020; Tapia-Rojo et al., 2019).

**Fig. 2.**
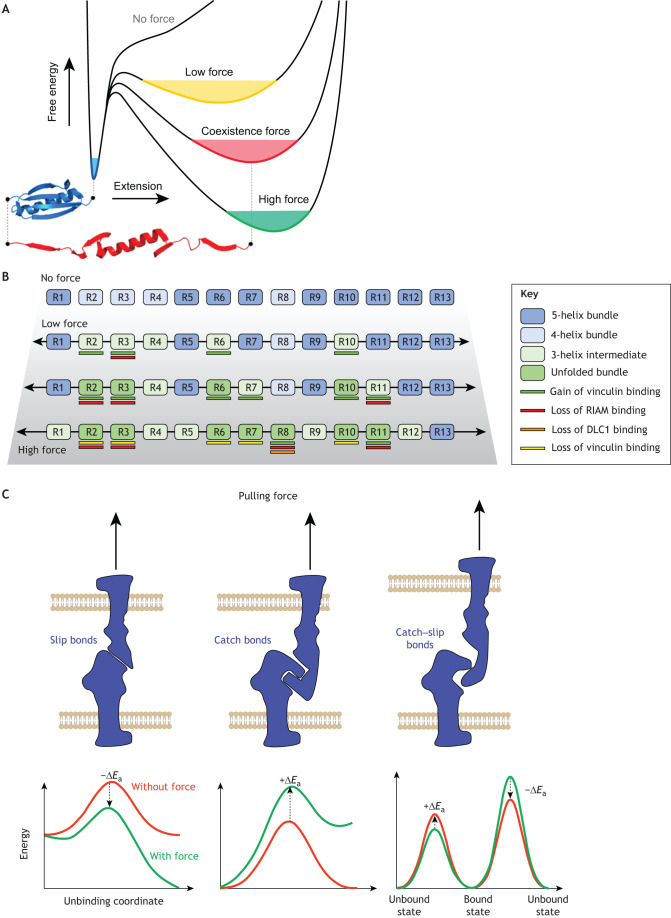
**Mechanical load applied on a protein modulates the free energy landscape of folding.** (A) Mechanoregulated proteins can be considered as mechanoswitches, adopting multiple metastable states. In the absence of mechanical load, a protein folds into a certain well-defined structure (blue); however, other conformational states can be populated upon mechanical load (shown in red). In the presence of force, these states can be energetically equally favourable, or even lower in terms of the free energy compared to the native state as illustrated by the free-energy landscape shown. At low force (yellow), thermodynamics still favours the relaxed (blue) conformation, but the probability for transitioning to the partially unfolded state (red) is already high. In such a landscape, a coexistence force condition (red) can exist, where the relaxed and partially unfolded states are both expected to be populated with similar probability. Finally, above a certain force threshold (green), the partially unfolded conformation is the energetically favoured state. At high force, the protein will become fully unfolded. (B) Talin has a highly complex free-energy landscape, as its 13 rod domains (R1–R13) all exhibit this switch-like behaviour in response to mechanical force. (C) The architecture of a bond defines its behaviour under mechanical load. Three different interaction mechanisms are illustrated. In the case of a slip bond (left), the lifetime of the bond decreases when mechanical load is applied and can be seen as a decrease of activation energy required for the dissociation. In the case of catch bond (middle), the bond lifetime increases under mechanical load, observed as an increase in the activation energy needed for the dissociation event. In the case of large biological macromolecules such as proteins, the bonds often display a catch–slip bond behaviour (right), in that they exhibit catch bond behaviour under low force, but increased force triggers slip-bond behaviour. In the case of the energy landscape for catch–slip bonds, two force-dependent unbinding pathways have to be considered: at low forces, the catch-bond behaviour is dominant, resulting in an increase in the activation energy (Ea) under applied load (unbinding to the left in the energy diagram), whereas at higher forces, the slip-bond behaviour leads to decrease in the activation energy (unbinding to the right). Panel A reprinted with permission from Stannard et al. (2021). Copyright 2021 American Chemical Society. Panel B reprinted from Mykuliak et al. (2018) where it was published under a CC-BY 4.0 license. Panel C reproduced from Huppa and Schütz (2016) with permission from Elsevier.

The most striking example of this mechanical load influence on protein conformation is on protein domains that can exist in distinct conformations, where switching between states can be triggered by mechanical force (Fig. 2). This complexity soon scales when there are multiple domains within a protein that can each adopt different low-energy conformations independently of each other, creating molecules that can have numerous different patterns or conformations dependent on the history of the forces that have acted on them (Goult, 2021) with different biological outcomes, illustrated schematically in Fig. 2B for the talin rod (Mykuliak et al., 2018; Yao et al., 2016).

#### Protein architecture

**Fig. 3.**
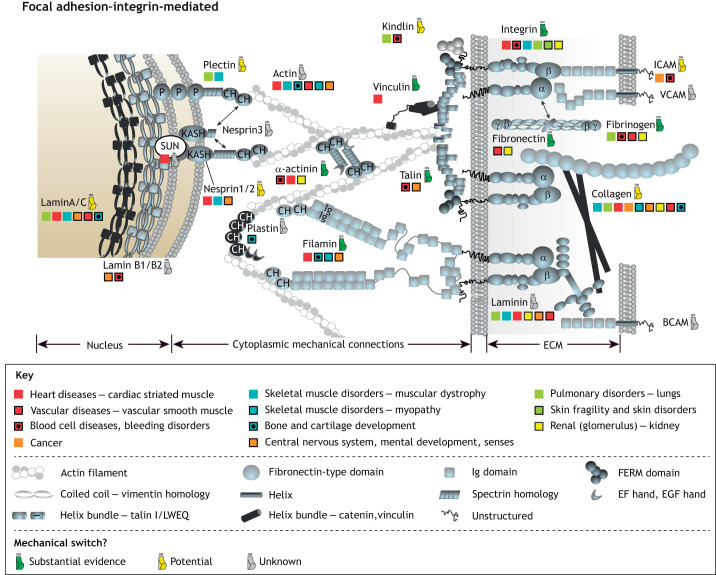
**The mechanical linkages between the extracellular matrix and the nucleus.** A simplified schematic of the linkages that a cell makes, via its integrin receptors, to the extracellular matrix (right). The ECM components, collagen, fibronectin, laminin and fibrinogen are shown, which are recognised by different integrin isoforms. Middle, on the cytoplasmic face of the integrin–ECM linkages, complex adhesive structures assemble, which are dynamic and mechanoresponsive. Left, the cytoskeletal connections emanating from the adhesion sites directly couple to nuclear envelope proteins, providing a direct mechanical coupling between the outside of the cell and the nucleus. Signals from the surface of the nucleus are propagated into the nucleus to alter the expression patterns and accessibility of genes. The association of the proteins with various diseases is indicated with the colour code as defined in the Key (top section). We stress that the disease associations presented are not exhaustive, particularly as new associations are being discovered all the time. However, even with this abridged dataset, the disease mappings clearly highlight the central role the mechanical machinery has in the correct functioning of the cell and how defects in the balance of these systems at the cellular level manifest as diverse disease states at the organismal level. Key: the protein domains involved for assembling mechanical linkages are defined in the key (middle section). Domains not indicated are as follows: CH, calponin homology; KASH, KASH domain; P, plectin homology. The symbols used are based on the structural features of the domains employing the structural data available in the Protein Data Bank (see also Box 2). The colour of the switch symbol for a protein (lower section) indicates the currently available evidence for mechanical switch properties. For details, please see Tables S1–S3.

**Fig. 4.**
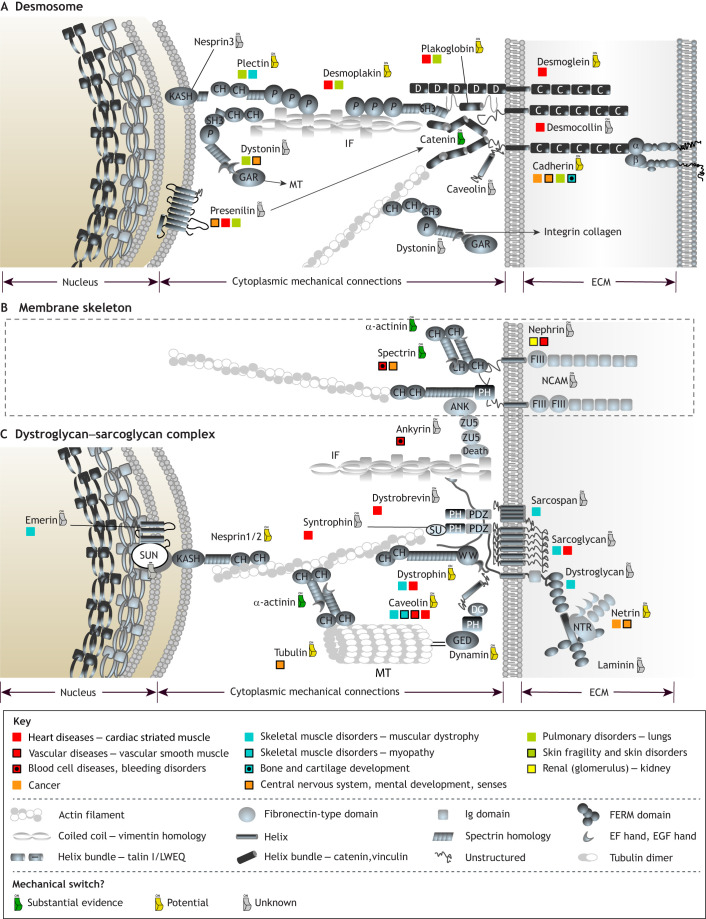
**Mechanical linkages the cell makes to the outside world.** (A) Desmosomes are specialised adhesive protein complexes responsible for maintaining the mechanical integrity of tissues. Complex protein networks link the ECM and neighbouring cells with the nucleus and cytoskeletal components. (B) The membrane skeleton is a specialised part of the cytoskeleton in close proximity of the cell membrane with a unique protein composition. (C) The dystroglycan–sarcoglycan complex forms a critical link between the cytoskeleton and ECM. The association of the proteins shown with various diseases is indicated with the colour code as defined in the Key (top section). Key: the protein domains involved for assembling mechanical linkages are defined in the key (middle section). Domains not indicated are as follows: ANK, ankyrin repeat; C, cadherin repeat; SU, calponin binding; Death, death domain; D, Desmoglein repeat; DG, dynamin type G domain; KASH, KASH domain; GED, GTPase effector domain; GAR, microtubule-binding domain; PDZ, PDZ domain; PH, Pleckstrin homology; P, plectin homology; SH3, SH3 domain; WW, WW domain; ZU5, ZU5 domain. IF, intermediate filaments; MT, microtubules. For details, please see Tables S1–S3.

It has been observed that all proteins are only marginally stable [Δ*G* being ∼−10 kcal/mol (Taverna and Goldstein, 2002; Williams et al., 2006)]. The relatively low stability might simply be an inherent property of the vast sequence space (Taverna and Goldstein, 2002), or it might be connected to the need for protein recycling; however, it could also reflect the requirement of conformational plasticity. Traditionally, studies of protein folding and stability focused on thermostability and stability against denaturing factors. More recently, the importance of the mechanostability of proteins has been appreciated. For example, a recent study proposed that an R495W mutation in cMyBP-C (encoded by *MYBPC3*), a myosin-associated protein located along the myosin thick-filament backbone in muscle (Bennett et al., 1986), decreases its mechanical stability and causes hypertrophic cardiomyopathy (Suay-Corredera et al., 2021). There are many such examples, discussed below, that together underline the relevance of these mechanical linkages in maintaining cellular mechanohomeostasis (Figs 3 and 4).

Therefore, an obvious question to ask is whether thermodynamic stability alone can predict the mechanical stability of a protein? The answer appears to be that it cannot. When proteins experience mechanical load, the system is tilted into a non-equilibrium state, and domain unfolding can only be prevented by bonds that are capable of resisting the applied mechanical load. As a result, coordinated interactions, namely hydrogen bonds, are much more important than hydrophobic contacts for the mechanical stability of a protein, whereas hydrophobic interactions are considered more important for the thermodynamic stability of proteins (Kellis et al., 1988; Pace et al., 2011). Comparison between proteins with equal thermostability but different secondary structure has shown that proteins with a lot of β-sheets have a much higher mechanical stability than α-helical proteins (reviewed in Carrion-Vazquez et al., 2000). This is relatively simple to explain by comparing the architecture of the secondary structures. In the case of a β-sheet, each polypeptide strand is almost completely extended, with extensive hydrogen bonding to the neighbouring strand. Therefore, it is more difficult to mechanically unfold β-sheets compared to α-helices, which can be unfolded bond-by-bond (Paci and Karplus, 2000). Furthermore, α-helices pack together mostly via hydrophobic interactions, causing them to be more vulnerable to local mechanical unfolding as compared to β-sheets, which are connected via hydrogen bonds throughout the structure. This is exemplified by the relative mechanical stabilities of β-sheet immunoglobulin (Ig) domains, which as measured by AFM is in the range of 50–300 pN (Oberhauser et al., 2002), whereas the typical unfolding force of a helix bundle measured by AFM is in the range of 10–30 pN (Haining et al., 2016).

It has been possible to identify ‘hot spots’ in proteins that contribute to enhanced mechanical stability, also referred to as ‘mechanical clamps’; these are often enabled by β-strands in shearing configuration, observed for example in titin (Sikora et al., 2009, 2011). These gatekeeper regions are capable of resisting high forces for a short period of time, so as to ensure protein stability under transient mechanical load (Craig et al., 2001). The reciprocal case also occurs where ‘weak spots’ in proteins will unfold first when the molecule experiences force (Yao et al., 2014a,b). The differences in unfolding kinetics of different regions of a multidomain protein might define the order of mechanical unfolding of the individual domains (Yao et al., 2016).

Another important mechanism is force buffering, where certain protein segments unfold rather easily, protecting the other parts of the protein from mechanical unfolding. An example of such a mechanism is the titin subdomain I27, which protects against disruption of the A-band organisation of the sarcomere during high-force load (Li et al., 2020). At a molecular level, titin I27 has been found to adopt an intermediate state that is largely independent of the applied load, potentially protecting the rest of the protein from unfolding (Nunes et al., 2010). A similar mechanism has been proposed for myomesin proteins, where α-helical linkers act as force-buffering motifs to maintain the integrity of the M-band of the sarcomere (Berkemeier et al., 2011). In talin, the unfolding of individual domains results in a decrease in tension and so the multiple domains unfolding and refolding as tension levels change help to maintain the applied load at <10 pN, enabling talin to buffer against large force changes (Yao et al., 2016). Another example of a multi-domain protein serving as ‘shock-absorber’ is dystrophin (Le et al., 2018), which protects the sarcolemma from damage against excess force via a similar mechanism.

#### Force-dependent interactions between biomolecules

As well as the force dependence of protein conformation, interactions between proteins are similarly susceptible to mechanical regulation. This can be explained with reference to the Arrhenius equation, which states that the lifetime of a bond is negatively dependent on temperature. This means that activation energy is needed to dissociate a bond, and so a mechanically loaded bond becomes shorter in life span. Such bonds are often referred to as ‘slip bonds’ and force exerted on two proteins binding in this way will weaken the interaction (Fig. 2C, left). In many cases, however, biological systems contain mechanisms that lead to the opposite situation – under mechanical load, the bond lifetime increases. Such behaviour is named ‘catch bond’, referring to fishing tools, and means that forces exerted on the interacting proteins strengthen the interaction (Fig. 2C, middle). Many interactions exhibit ‘catch–slip bond’ behaviour, where forces initially increase the interaction and lifetime of the bond, but higher forces weaken the bond (Fig. 2C, right).

Catch bonds are important for the force-generation machinery of the cell, and new examples are being reported regularly. Again, talin offers an intriguing example – its actin-binding site 3 (ABS3) interacts with F-actin only weakly in biochemical assays (Gingras et al., 2008). When optical tweezers were used to apply mechanical load on the talin–actin bond *in vitro* it was observed that the binding lifetime was >100-fold longer when pulled towards the pointed end of the actin filament compared to when applied towards the barbed end of the filament (Owen et al., 2022). This directional catch bond is similar to catch bond between vinculin and actin (Huang et al., 2017), and this directionality appears to be due to the inherent polarity of the actin filament (Swaminathan et al., 2017). Another example of a catch bond is observed in the microtubule-associated motor protein dynein family, where an individual dynein on its own is capable of generating rather low force (∼1 pN) (Mallik et al., 2004). However, under high force, dyneins form catch bonds that bind microtubules tightly (Kunwar et al., 2011; Leidel et al., 2012) and thus are able to withstand higher force by varying step size (Rai et al., 2013). These features enable dyneins to generate large collective forces in cells (Mallik et al., 2004).

#### Mechanochemical switches – quantising responses to force and protecting against thermal noise

The cell is a busy, dynamic place, and amidst the milieu of proteins, chemical inputs and mechanical cues a cell experiences it could appear almost chaotic. However, in among all this ‘hustle and bustle’, the cell can use these mechanical inputs in a meaningful way. One way that the cell can achieve this is to use ‘mechanical switches’, domains that change their structure, function or role as a function of force (Box 1).

**Box 1. Requirements for a good mechanical switch**

When mechanical stimuli above a certain level are met, a mechanical switch can change state, leading to more persistent changes in the signalling at this site. In this way, a switch can experience lots of small force changes, but its output is quantised in that it is either in one state or the other. The output of such a process could be, for example, altered ligand binding or posttranslational modification. Furthermore, these switches offer the possibility of ‘logic gates’ as force above a certain threshold AND a certain ligand gives one response, whereas force above a certain threshold AND an active enzyme gives a different response (described in Goult et al., 2021). Here, AND indicates a logic gate having two or more inputs and a single output. Below, we detail key features of a mechanical switch as we believe that these switches play a critical role in mechanobiology.

A mechanical switch needs to:

**(1) have domains that can alter their conformations reversibly.** Mechanosensitive protein domains have two or more conformations which differ in length in the absence or presence of force (Wang et al., 2019b).

**(2) reset when force is removed.** The ideal scenario is that the switch has two (or more) thermodynamically stable states, and that these can be toggled between using small changes in mechanical force.

**(3) be part of a mechanical linkage**. Or be tethered in some way that allows it to experience and measure force, either exposed to a moving part, or in a moving current.

**(4) be able to alter in response to force**. This force might be generated by the cell moving, or by alterations in shape, or stress, or it might be actively generated by the cytoskeletal machinery, for example, by actin retrograde flow, or the myriad motor proteins that generate motion in the cell. Polymerisation of long filaments is another way to generate forces.

Another important feature of the switches identified to date is that they exhibit ‘mechanical hysteresis’, whereby the force required to unfold a domain is significantly higher than the force at which that domain will refold (Yao et al., 2016).

Such switches are present in all of the mechanical linkages identified to date, with more being discovered regularly. Proteins such as fibronectin in the ECM (Peleg et al., 2012), talin and vinculin in focal adhesions (FAs), α-catenin (Yao et al., 2014b) and dystrophin (Le et al., 2018) at the intracellular side of cell membrane, nesprin (Déjardin et al., 2020) and lamin A/C (Cho et al., 2019) at the nuclear envelope all contain mechanical switches (Fig. 1). The mechanosensitive protein talin represents a particularly complex molecule in this regard, as it exhibits mechanosensitivity through the helical bundles in its rod domains that act as switches (del Rio et al., 2009; Haining et al., 2016; Hytönen and Vogel, 2008; Vigouroux et al., 2020; Yao et al., 2016). As talin contains 13 of these mechanochemical switches in its rod domains, R1–R13 (Goult et al., 2013), each with the ability to fold and refold repeatedly and with high fidelity (Goult et al., 2021; Yao et al., 2016), the switch patterns talin can adopt are complex. A common feature of these rod domains is that they contain, buried within their hydrophobic core, polar residues (Ser/Thr) that tune their mechanical stabilities (Goult et al., 2013; Han et al., 2021; Rahikainen et al., 2017). Some of these residues have been identified as phosphorylation sites in proteomic studies (Bian et al., 2014; Mertins et al., 2016; Ratnikov et al., 2005), and a striking possibility is that, once exposed, these residues are phosphorylated, which would prevent domain refolding. These switches, built into the meshwork of adhesion and cytoskeletal proteins, can be viewed as a type of code, a MeshCODE (Barnett and Goult, 2022 preprint; Goult, 2021), where the pattern of binary information (folded ‘0’ and unfolded ‘1’) encoded in the shape of these molecules provides instructions to dynamically respond to changes in mechanical forces the cell experiences altering the signalling of that adhesion and that cell (Goult et al., 2018). A switch unfolding also introduces a quantised step-change in the length of the talin molecule (40–120 nm depending on the switch) altering the spatial organisation of molecules in the linkages (Barnett and Goult, 2022 preprint). Similar switches have been identified in many other mechanosensitive proteins distributed throughout the cell (Figs 1, 3 and 4). As these switches are all coupled to the cytoskeleton, this indicates a mechanism for how they might work cooperatively allowing the entire cell to function as a mechanical machine forming long-range interdependencies that synchronise cellular operations (Figs 1, 3 and 4).

#### Cryptic binding sites

One of the best-known examples of mechano-regulated binding is the association of vinculin with target proteins. Early studies showed that local mechanical manipulation of a cell led to subcellular accumulation of vinculin (Balaban et al., 2001; Riveline et al., 2001). Structural studies provided the first hint of a mechanism for this force-dependent interaction when it was found that the vinculin-binding sites (VBSs) in talin were buried inside the folded talin bundles (Gingras et al., 2005; Izard et al., 2004; Papagrigoriou et al., 2004), suggesting talin domains might need to unfold to enable vinculin binding. Later, single-molecule pulling experiments *in vitro* (del Rio et al., 2009; Yao et al., 2016) and in computational simulation (Hytönen and Vogel, 2008) revealed that mechanical forces can cause talin rod domains to transition from a folded-bundle arrangement in its relaxed state, in which the VBS is cryptic, to an extended conformation that gradually exposes the amino acids of the VBS in the helices and facilitates vinculin binding. Vinculin binding limits domain refolding but mechanically stabilises the talin VBS helix, as shown by stretching talin rod domains in the presence of bound vinculin. Dissociation of vinculin is immediately followed by elongation of talin, as the VBS transitions from a helix to a random coil (Yan et al., 2015), an effect that can be used to calculate the force-dependent binding constant (Wang et al., 2019b, 2021). Vinculin binding is reversible – once relaxed, the talin domains refold and vinculin molecules are released and free vinculin adopts an autoinhibited conformation (Cohen et al., 2006). However, if vinculin autoinhibition is disrupted, either by using just its head domain (Bakolitsa et al., 2004) or mutated forms of vinculin lacking autoinhibition (Cohen et al., 2005), some of the complexes formed with talin are not released (Carisey et al., 2013; Wang et al., 2021; Yao et al., 2014a, 2016). It has been observed that relief of autoinhibition also can lead to complex formation between talin and vinculin without mechanical load (Atherton et al., 2020; Kelley et al., 2020), but this is because autoinhibition itself is a mechanosensitive phenomenon (Khan and Goult, 2019). Constitutively active vinculin is lethal in flies (Maartens et al., 2016) and causes large adhesions in cells that do not disassemble efficiently, limiting cell migration (Carisey et al., 2013). It has been observed that only a small fraction (<15%) of talin proteins are stretched *in vivo* in *Drosophila* muscle-attachment sites (Lemke et al., 2019). This might indicate that the cellular mechanosensing machinery maintains a delicate balance in the amount of mechanical load applied to individual molecules.

The VBSs within other interaction partners, such as α-actinin and α-catenin proteins, also become exposed under mechanical load (Bois et al., 2005; Le et al., 2017; Yao et al., 2014b). Vinculin interactions are not the only examples of mechanical exposure of cryptic sites. By definition, all autoinhibited proteins contain cryptic binding sites (Pufall and Graves, 2002), and if those proteins form mechanical linkages, these will be force-dependent cryptic binding sites, as mechanical load will stabilise the open conformation (Khan and Goult, 2019). Examples include the activities of focal adhesion kinase (FAK; also known as PTK2) (Bauer et al., 2019) and titin kinase (Gräter et al., 2005), which are both modulated by mechanical signals. The ECM contains multiple examples of mechanically regulated interactions that help organise the meshworks. For example, fibronectin contains binding sites for collagen (Kubow et al., 2015) and multiple cryptic self-association sites (Lemmon and Weinberg, 2017), which are activated under mechanical load and are important for fibronectin fibrillogenesis. Similarly, collagen fibres also contain mechanically adjustable binding sites (Zhu et al., 2018).

It seems likely that there are many more binding sites where the accessibility is mechanically regulated in the protein interaction networks of the cell to be discovered. Learning more about the mechanoregulation of protein conformation and regulation of protein interactions might help in understanding the molecular basis of diseases associated with mechanically coupled proteins (Figs 3 and 4).

#### Post-translational regulation of mechanical switch domains

PTMs modulate the stability, interactions, localisation and conformations of proteins. This raises the question of whether PTMs also contribute to their mechanical stability? Or alternatively, could mechanical signals alter PTMs within proteins?

Recent studies have revealed that cyclin-dependent kinase 1 (CDK1), a key regulator of the cell cycle, contributes to adhesion dynamics (Jones et al., 2018) in part via phosphorylation of talin (Gough et al., 2021). Phosphorylation of talin (at residue S1589 in the R7–R8 linker) by CDK1 leads to alterations in the order of how the talin rod domains unfold, therefore modulating the mechanical response of talin (Gough et al., 2021).

Talin also contains a force-dependent calpain cleavage site in the R10 switch (Zhang et al., 2012); force exerted on talin AND active calpain leads to cleavage [an example of an ‘AND’ gate operation (see Box 1), as force alone ‘OR’ calpain activity alone generate different outcomes (Bate et al., 2012)]. It is likely that the proteolytic events associated with talin and related molecules are regulated by mechanical load to yield different processed versions, as calpain cleavage between the talin head and rod is required for proper cell adhesion (Saxena et al., 2017), and similarly, talin rod cleavage is important for correct adhesion dynamics (Bate et al., 2012).

Force-regulated proteolytic cleavage is also important in many other biological systems. For example, for von Willebrand factor (VWF), a large multimeric protein found in blood plasma that mediates the adhesion of platelets to the connective tissue (reviewed in Lenting et al. 2012), it was found that mechanical load applied to VWF due to shear flow exposes a cleavage site within its A2 domain that allows for proteolytic activity by the metalloprotease ADAMTS13 (Baldauf et al., 2009). Similarly, in the protein recycling machinery, AAA+ proteases utilise mechanical force to unfold their target proteins (reviewed in Baker and Sauer, 2012). Interestingly, protein ‘knots’ exist that provide resistance against this mechanically assisted unfolding and proteolysis (Sriramoju et al., 2020) and >1300 knotted proteins have been identified (Dabrowski-Tumanski and Sulkowska, 2017).

Overall, mechanical load regulates protein conformation, and as many of the protein complexes discussed here are connected via mechanical linkages, mechanical load applied on one cellular component can impact on other connected structures. Therefore, to understand cellular mechanosignalling, it is important to decipher the mechanical force propagation pathways in cells.

## Cells as a mechanically linked machinery

In this section, we describe how the molecular building blocks described above are assembled into complex mechanical linkages that connect all parts of the cell and link to its immediate surroundings. Owing to the complexity of the cellular networks, it is challenging to visualise physical connections with sufficient detail, but visualisation of these networks is instructive for conceptualising how cells might function. Similarly, as with most of biology, incredible complexity is borne from simple components and rules, and the mechanical linkages in cells are constructed from a toolbox of protein domains with specific functionalities that are repurposed for different purposes (Box 2; Table S1). The numerous linkages have a modular composition, and we use illustrations that all employ a standardised structure code format to visualise the modular architecture of many of the cytoskeletal proteins, with the aim to build a structure–function picture of the mechanical connections of a cell (Figs 3 and 4).

**Box 2. Protein components to build a mechanical cell**

To visualise the mechanical apparatus of the cell we amalgamated the available information from the literature for the reported protein associations in these cellular assemblies, including the cellular location and known interactions, tissue specific expression, disease association, structural homology and genome information. This information was obtained from multiple databases including NCBI, PubMed, UniProt, Ensembl, PDB. The diseases listed were retrieved from NCBI OMIM (Online Mendelian Inheritance in Man) (Amberger et al., 2019). The vast majority of diseases shown are a result of DNA mutation (missense, frameshift) and although changes in expression levels of proteins also correlate with disease, these were not considered in our analysis. The information sources are shown in Table S3.

Information on the contribution of mechanical signals in disease is rapidly expanding, but there is still much to understand. In Figs 3 and 4, we have illustrated a number of the cellular mechanical linkages, where the protein components were selected using the following criteria: (1) the protein of interest is **mechanically active**, or affected by **mechanical cues**; (2) alternatively, the target protein **interacts** with a mechanically active protein; (3) its cellular location potentially contributes to the **transmission of mechanical signals** between cellular structures; (4) it has known disease association; (5) there is structural information available.

The visualisation is based on known domains, and molecular networks shown in Figs 3 and 4 are built using the domains as described in the Key and legends.

Actin filaments that dynamically connect cellular substructures are crucial in mediating mechanical signals and producing mechanical load via molecular motors (myosin) (Houdusse and Sweeney, 2016). Similarly, the microtubule (Hamant et al., 2019) and intermediate filament (IF) (van Bodegraven and Etienne-Manneville, 2021) cytoskeletons also form connections and act as sensors for mechanical load. This network of cytoskeletal filaments transmits mechanical cues across the cell enabling changes in the physical environment to be rapidly transferred to each cellular compartment. The cytoskeleton directly couples to the nuclear envelope, via nesprin and SUN proteins (Lombardi et al., 2011) of the LINC (linker of nucleoskeleton to cytoskeleton) complex (Crisp et al., 2006). These direct couplings to the nucleus enable force-dependent alterations in gene expression (Cooper and Giancotti, 2019; Elosegui-Artola et al., 2016; Engler et al., 2006; Graham and Burridge, 2016; Jahed et al., 2014; Jain et al., 2013). While it is likely that many of the proteins involved in these networks are acting as mechanical switches, only a limited number of switches are characterised (Table S2), as indicated in Figs 3 and 4.

### Mechanical connections within cells and their association with diseases

Not surprisingly, maintaining the correct level of physical cues is essential to tissue integrity and to health and so cellular mechanosignalling must involve powerful mechanisms that regulate the force-generating cellular machineries to work in synchrony to maintain homeostasis as the physical properties of the environment changes (termed mechanohomeostasis). We are in the early phase of understanding the mechanoregulation of cellular processes, although disturbed mechanohomeostasis is being increasingly linked to pathological conditions (highlighted in the recent editorial ‘Pathological mechanosensing’; see https://www.nature.com/articles/s41551-021-00835-5).

Typical examples of diseases associated with defects in mechanosignalling are cancer (Schedin and Keely, 2011) and fibrosis (Tschumperlin et al., 2018). However, links between defects in mechanotransduction and cardiomyopathy (Clippinger et al., 2019), atherosclerosis (Irons and Humphrey, 2020) and osteoporosis (Haffner-Luntzer et al., 2020), have now been identified and there is a rapidly expanding amount of genetic information on diseases that result from defects in these linkages. To that end, we have collated information about diseases potentially linked to defects in mechanotransduction and mapped them onto the schematic representations of these linkages (Figs 3 and 4; Table S3). Together they provide insight into how dysregulated operation of this machinery can give rise to disease.

Some disease-causing mutations impact multiple linkages. For example, mutations in actin, which manifest in diseases such as cardiomyopathy (Olson et al., 1998), will perturb each linkage. Similarly, each linkage involves coupling to the nucleus, where lamin proteins have a significant role in enabling the proper mechanoresponse (Ihalainen et al., 2015; Swift and Discher, 2014). Laminopathies are a diverse group of diseases associated with mutations in A-type lamins (reviewed in Davidson and Lammerding, 2014) and will impact all linkages. However, other mutations specifically disrupt certain linkages.

#### Linkage 1 – integrin–adhesion complexes, connecting cells to the ECM

The FA is one of the best studied mechanical linkages connecting the ECM to the cytoskeleton, downstream signalling pathways, and to the nucleus via direct linkages to the LINC complex (Fig. 3). The core of FAs involves integrins attached to the ECM (Bachmann et al., 2019) coupled to the cytoskeleton via talin and vinculin, and >250 additional components of the integrin ‘adhesome’ (Chastney et al., 2020; Horton et al., 2015; Winograd-Katz et al., 2014) can assemble onto this core to form integrin signalling complexes. FAs are mechanoresponsive and change their composition as a response to applied mechanical load (Kuo, 2013).

Mutations in ECM proteins are common in diseases, such as epidermolysis bullosa, affecting collagen (Varki et al., 2007), and muscular dystrophies, that is with mutations in laminin (Helbling-Leclerc et al., 1995). In collagens alone, >1000 disease-associated mutations have been reported (Myllyharju and Kivirikko, 2001). Numerous diseases are associated with integrin mutations, including muscular dystrophies and skin blistering (reviewed in Bouvard et al., 2001). On the cellular side, mutations in integrin adapter proteins, such as filamin, cause diseases including skeletal dysplasia and intestinal obstruction (reviewed in Sasaki et al., 2019). Talin mutations have been linked to multifaceted clinical symptoms (Azizi et al., 2022), cancer (Azizi et al., 2021) and spontaneous coronary artery dissection (Turley et al., 2019). There are growing numbers of diseases recognised as being associated with building blocks of these linkages and a better understanding of the role of mechanical signals in the regulation of cell–ECM interactions should enable development of novel therapeutic applications and strategies (reviewed in Winograd-Katz et al., 2014). Such approaches include targeting specific integrins (Bachmann et al., 2019; Bergonzini et al., 2022) or the downstream signalling pathways (Pang et al., 2021) in the treatment of cancer (Cooper and Giancotti, 2019).

#### Linkage 2 – the desmosome, connecting cells and cytoskeleton

Desmosomes (Fig. 4A) represent mechanically strong cell–cell adhesion structures that are often found in tissues experiencing high mechanical load, such as cardiac tissue and bladder (Delva et al., 2009). Arrhythmogenic cardiomyopathy is commonly associated with defects in desmosomes and often caused by mutations in desmoglein or desmocollin (Lahtinen et al., 2011), which are transmembrane desmosomal proteins responsible for the cell–cell interactions. However, there are also diseases associated with the intracellular constituents of desmosome (Fig. 4A).

For example, mutations in desmoplakin are associated with diseases reflecting compromised tissue integrity under mechanical load, such as cardiomyopathy (Norgett et al., 2000) and skin fragility (Whittock et al., 2002). Using genetically encoded Förster resonance energy transfer (FRET) mechanoreporters, it has been shown that desmoplakin experiences little or no load resulting from internally generated contractile forces, but externally applied mechanical forces resulted in mechanical load on desmoplakin suggesting that desmosomes function as stress-absorbing adhesion complexes (Price et al., 2018). Dystonin (also known as bullous pemphigoid antigen-1) connects IFs and microtubules (Bouameur et al., 2014), and mutations in dystonin have been linked to neurological disorders and skin blistering (Groves et al., 2010). Via IFs, plectins link desmosomes to the nucleus, and mutations in plectin have been linked to epidermolysis bullosa simplex with muscular dystrophy (Bardhan et al., 2020; Pfendner et al., 2005). Another example of a protein that connects desmosomes to the nucleus is presenilin. Interestingly, presenilin has also been observed to be physically connected to catenin (Yu et al., 1998) at adherens junctions, highlighting the crosstalk and interdependencies of these linkages, and mutations in presenilin are associated with heart diseases (Li et al., 2006) and Alzheimer's disease (Hutton and Hardy, 1997).

#### Linkages 3 and 4 – the membrane skeleton and the dystrophin glycoprotein complex

The membrane skeleton refers to a specialised part of the cytoskeleton that is in close proximity to the cell membrane and differs from the bulk cytoskeleton in its protein composition and structure (Ritchie and Kusumi, 2004). The membrane skeleton (Fig. 4B) is formed of a network of spectrin and actin (Bennett and Baines, 2001), and is important in preserving the integrity and mechanical characteristics of the cell membrane. Spectrin mutations are associated with anemia and neurodegenerative diseases (Li et al., 2022).

The dystrophin glycoprotein complex (DGC, Fig. 4C) is another membrane-spanning complex linking the ECM to the cytoskeleton and mechanically coupling it to the nucleus (Ibraghimov-Beskrovnaya et al., 1992), especially in cardiac and skeletal muscle (Lapidos et al., 2004). Dystroglycan is a non-integrin ECM receptor that is linked to cytoplasmic actin filaments via the mechanosensitive protein dystrophin, and DGC is involved in multiple processes, including basement membrane assembly, nerve myelination and epithelial polarisation. The sarcoglycan complex is a subcomplex within the DGC (Hack et al., 2000). Sarcoglycanopathies are muscular dystrophies caused by mutations in any of the four sarcoglycan proteins (Fayssoil, 2010). Mutations in δ-sarcoglycan can influence DGC function causing myocardial mechanical instability (Campbell et al., 2016). Mutations and deletions in the central domain of dystrophin are linked to a large number of mild skeletal muscle disease cases, as well as to severe cardiomyopathy. Via the actin cytoskeleton, DGC connects to the nucleus via nesprins, and nesprin-1 mutations are associated with dilated cardiomyopathy and cause disruption of nuclear envelope (Zhou et al., 2017), highlighting how defects in different parts of mechanical linkages can manifest in similar pathologies. Furthermore, deletions in the central domain of dystrophin are observed in patients with late-onset Becker muscular dystrophy (Bushby et al., 1993). Point mutations that influence the stability of dystrophin are also associated with diseases; for example, an L427P mutation results in partial misfolding and reduced rate of refolding of the spectrin-like domains 1–2 of the 24 present (Acsadi et al., 2012), indicating altered mechanical stability. Another protein connecting to the dystroglycan receptor is caveolin, which coordinates the anchorage of caveolae (Sharma et al., 2010) but is also a scaffolding protein associated with a number of diseases (Cohen et al., 2004) (Fig. 4B). Caveolae are small invaginations of the plasma membrane involved in processes such as endocytosis (Hetmanski et al., 2019).

These examples indicate how mechanical connections between cells are essential for tissue function, and even small disturbances in these linkages transmitting mechanical force can cause significant defects. Therefore, as information about genetic factors increases it will be important to take mechanical signalling into account when trying to understand the molecular mechanisms underlying diseases.

## Conclusions and perspectives

Mechanical linkages provide a way to coordinate long-range activities in a cell and to harness the force-generation and force-sensory machinery to control cell behaviour. Together, this leads to the emergence of a view of a mechanical cell with patterns of switch states that define its overall behaviour. The integration of an array of binary switches distributed across each cell into these sensory networks indicates a way each cell could achieve exact states and quantised responses. As each cell is connected to its neighbours and the ECM, interdependencies between these linkages enable biological systems to maintain their operation and synchronise behaviours across multiple levels of complexity in a way that is metastable and robust. We envisage that the switches outside of the cell, such as conformations of ECM proteins, affect the signalling inside the cell and reciprocally, that the switches inside the cell alter the mechanical load exerted on the ECM. This enables precise synchronisation between the ECM and the cell, which is difficult to recapitulate in cell culture. In the case of multimodular proteins, applied mechanical load might adjust the biological function by influencing the conformation and activity of multiple domains simultaneously (Goult et al., 2021). While force-resistant domains of the protein remain folded, force-sensitive domains change their function under mechanical stress. Therefore, multimodular proteins, which represent the majority of human proteins (Ekman et al., 2005), might perform an array of different functions that are modulated by chemical and physical cues (Vogel, 2006).

Furthermore, mechanical regulation of proteins represents an as-yet untapped therapeutic opportunity; targeted therapeutic molecules that adjust the mechanical stability of mechanosensors could be used to alter the behaviour of an entire cell, but development of such drugs requires a deeper understanding of molecular behaviour. Information about protein interaction networks might represent a novel way to consider diseases in the context of mechanosignalling, and viewing such diseases as a disruption of these mechanical networks might pave the way for advances in personalised medicine. Identification of a *de novo* mutation in a protein from a patient presenting with a condition might map to a mechanical linkage and thus identify existing ways to treat that defect.

We are still in the early stages of understanding the mechanoregulation of cellular functions. Although the behaviour of individual proteins under mechanical load has been studied, we have a significant knowledge gap regarding the regulation of protein interactions and functional alterations by mechanical signals throughout cellular networks. With this Review, we encourage researchers and clinicians to pay attention to mechanical connections and their potential contribution to disease.

## Supplementary Material

10.1242/joces.259769_sup1 Supplementary information
