# Supplementary material for: The mechanical cell – the role of force dependencies in synchronising protein interaction networks
Source: J Cell Sci. 2022 Nov 18;135(22):jcs259769. doi: 10.1242/jcs.259769 (PMC9845749; doi:10.1242/jcs.259769)
Supplement: Supplementary information [file joces-135-259769-s1.pdf]

Table S1. Structural features of proteins

Click here to download Table S1

Table S2. Mechanical switches

Protein	Considered as a switch (Yes/Likely/Unknown)	Reference, preprint doi
Actin	Unknown	(De La Cruz and Gardel, 2015)
α-actinin	Yes	(Le et al., 2017)
Ankyrin	Unknown	
BCAM	Unknown	
Cadherin	Likely	(Borghi et al., 2012; Conway et al., 2013)
Catenin	Yes	(Ishiyama et al., 2013; Yao et al., 2014)
Caveolin	Unknown	(Moreno-Vicente et al., 2018)
Collagen	Likely	(Ames et al., 2016)
Desmocollin	Unknown	
Desmoglein	Likely	(Baddam et al., 2018; Uttagomol et al., 2019)
Desmoplakin	Likely	(Daday et al., 2017; Price et al., 2018)
Dynamin	Likely	(Danino and Hinshaw, 2001)
Dystonin	Unknown	
Dystrobrevin	Unknown	
Dystroglycan	Unknown	
Dystrophin	Likely	(Le et al., 2018)
Emerin	Unknown	
Fibrinogen	Yes	(Butera and Hogg, 2020)
Fibronectin	Yes	(Ingham et al., 2004; Klotzsch et al., 2009; Smith et al., 2007; Zhong et al., 1998)
Filamin	Yes	(Huelsmann et al., 2016)
ICAM	Likely	(Liu et al., 2010), https://doi.org/10.1101/2020.06.29.177816
Integrin	Yes	(Friedland et al., 2009)
Kindlin	Likely	(Jahed et al., 2019)
Lamin A/C	Likely	(Cho et al., 2019)
Lamin B1/B2	Unknown	(Vahabikashi et al., 2022)
Laminin	Unknown	
NCAM	Unknown	
Nephrin	Unknown	
Nesprin 3	Unknown	
Nesprin 1/2	Likely	(Déjardin et al., 2020)
Netrin	Likely	(Moore Dr. et al., 2012)
Plakoglobin	Likely	https://doi.org/10.1101/2022.03.13.484158
Plastin	Unknown	
Plectin	Likely	(Almeida et al., 2015; Suman et al., 2019)
Presenilin	Unknown	
Sarcoglycan	Unknown	
Sarcospan	Unknown	
Spectrin	Yes	(Daday et al., 2017; Law et al., 2003; Moe and Cembran, 2020; Renn et al., 2019)
Sun	Unknown	(Donnaloja et al., 2019)
Syntrophin	Unknown	
Talin	Yes	See refs in main text
Tubulin	Likely	(Nasrin et al., 2021)
VCAM	Unknown	
Vinculin	Yes	(Grashoff et al., 2010)

Table S3. Disease associations

Click here to download Table S3

References

Almeida, F. V., Walko, G., McMillan, J. R., McGrath, J. A., Wiche, G., Barber, A. H. and Connelly, J. T. (2015). The cytolinker plectin regulates nuclear mechanotransduction in keratinocytes. *J. Cell Sci.* **128**, 4475–4486.

Ames, J. J., Contois, L., Caron, J. M., Tweedie, E., Yang, X., Friesel, R., Vary, C. and Brooks, P. C. (2016). Identification of an endogenously generated cryptic collagen epitope (XL313) that may selectively regulate angiogenesis by an integrin yes-associated protein (YAP) mechano-transduction pathway. *J. Biol. Chem.* **291**, 2731–2750.

Baddam, S. R., Arsenovic, P. T., Narayanan, V., Duggan, N. R., Mayer, C. R., Newman, S. T., Abutaleb, D. A., Mohan, A., Kowalczyk, A. P. and Conway, D. E. (2018). The desmosomal cadherin desmoglein-2 experiences mechanical tension as demonstrated by a FRET-based tension biosensor expressed in living cells. *Cells* **7**,.

Borghi, N., Sorokina, M., Shcherbakova, O. G., Weis, W. I., Pruitt, B. L., Nelson, W. J. and Dunn, A. R. (2012). E-cadherin is under constitutive actomyosin-generated tension that is increased at cell-cell contacts upon externally applied stretch. *Proc. Natl. Acad. Sci. U. S. A.* **109**, 12568–12573.

Butera, D. and Hogg, P. J. (2020). Fibrinogen function achieved through multiple covalent states. *Nat. Commun.* **11**,.

Cho, S., Vashisth, M., Abbas, A., Majkut, S., Vogel, K., Xia, Y., Ivanovska, I. L., Irianto, J., Tewari, M., Zhu, K., et al. (2019). Mechanosensing by the Lamina Protects against Nuclear Rupture, DNA Damage, and Cell-Cycle Arrest. *Dev. Cell* **49**, 920-935.e5.

Conway, D. E., Breckenridge, M. T., Hinde, E., Gratton, E., Chen, C. S. and Schwartz, M. A. (2013). Fluid shear stress on endothelial cells modulates mechanical tension across VE-cadherin and PECAM-1. *Curr. Biol.* **23**, 1024–1030.

Daday, C., Kolšek, K. and Gräter, F. (2017). The mechano-sensing role of the unique SH3 insertion in plakin domains revealed by Molecular Dynamics simulations. *Sci. Rep.* **7**,.

Danino, D. and Hinshaw, J. E. (2001). Dynamin family of mechanoenzymes. *Curr. Opin. Cell Biol.* **13**, 454–460.

De La Cruz, E. M. and Gardel, M. L. (2015). Actin mechanics and fragmentation. *J. Biol. Chem.* **290**, 17137–17144.

Déjardin, T., Carollo, P. S., Sipieter, F., Davidson, P. M., Seiler, C., Cuvelier, D., Cadot, B., Sykes, C., Gomes, E. R. and Borghi, N. (2020). Nesprins are mechanotransducers that discriminate epithelial-mesenchymal transition programs. *J. Cell Biol.* **219**,.

Donnaloja, F., Jacchetti, E., Soncini, M. and Raimondi, M. T. (2019). Mechanosensing at the nuclear envelope by nuclear pore complex stretch activation and its effect in physiology and pathology. *Front. Physiol.* **10**,.

Friedland, J. C., Lee, M. H. and Boettiger, D. (2009). Mechanically activated integrin switch controls α5β 1 function. *Science (80-.).* **323**, 642–644.

Grashoff, C., Hoffman, B. D., Brenner, M. D., Zhou, R., Parsons, M., Yang, M. T., McLean, M. A., Sligar, S. G., Chen, C. S., Ha, T., et al. (2010). Measuring mechanical tension across vinculin reveals regulation of focal adhesion dynamics. *Nature* **466**, 263–266.

Huelsmann, S., Rintanen, N., Sethi, R., Brown, N. H. and Ylänne, J. (2016). Evidence for the mechanosensor function of filamin in tissue development. *Sci. Rep.* **6**,.

Ingham, K. C., Brew, S. A. and Erickson, H. P. (2004). Localization of a cryptic binding site for tenascin on fibronectin. *J. Biol. Chem.* **279**, 28132–28135.

Ishiyama, N., Tanaka, N., Abe, K., Yang, Y. J., Abbas, Y. M., Umitsu, M., Nagar, B., Bueler, S. A., Rubinstein, J. L., Takeichi, M., et al. (2013). An autoinhibited structure of α-catenin and its implications for vinculin recruitment to adherens junctions. *J. Biol. Chem.* **288**, 15913–15925.

Jahed, Z., Haydari, Z., Rathish, A. and Mofrad, M. R. K. (2019). Kindlin Is Mechanosensitive: Force-Induced Conformational Switch Mediates Cross-Talk among Integrins. *Biophys. J.* **116**, 1011–1024.

Klotzsch, E., Smith, M. L., Kubow, K. E., Muntwyler, S., Little, W. C., Beyeler, F., Gourdon, D., Nelson, B. J. and Vogel, V. (2009). Fibronectin forms the most extensible biological fibers displaying switchable force-exposed cryptic binding sites. *Proc. Natl. Acad. Sci. U. S. A.* **106**, 18267–18272.

Law, R., Carl, P., Harper, S., Dalhaimer, P., Speicher, D. W. and Discher, D. E. (2003). Cooperativity in forced unfolding of tandem spectrin repeats. *Biophys. J.* **84**, 533–544.

Le, S., Hu, X., Yao, M., Chen, H., Yu, M., Xu, X., Nakazawa, N., Margadant, F. M., Sheetz, M. P. and Yan, J. (2017). Mechanotransmission and Mechanosensing of Human alpha-Actinin 1. *Cell Rep.* **21**, 2714–2723.

Le, S., Yu, M., Hovan, L., Zhao, Z., Ervasti, J. and Yan, J. (2018). Dystrophin As a Molecular Shock Absorber. *ACS Nano* **12**, 12140–12148.

Liu, Z., Sniadecki, N. J. and Chen, C. S. (2010). Mechanical forces in endothelial cells during firm adhesion and early transmigration of human monocytes. *Cell. Mol. Bioeng.* **3**, 50–59.

Moe, S. J. and Cembran, A. (2020). Mechanical Unfolding of Spectrin Repeats Induces Water-Molecule Ordering. *Biophys. J.* **118**, 1076–1089.

Moore Dr., S. W., Zhang, X., Lynch, C. D. and Sheetz, M. P. (2012). Netrin-1 Attracts axons through FAK-dependent mechanotransduction. *J. Neurosci.* **32**, 11574–11585.

Moreno-Vicente, R., Pavón, D. M., Martín-Padura, I., Català-Montoro, M., Díez-Sánchez, A., Quílez-Álvarez, A., López, J. A., Sánchez-Álvarez, M., Vázquez, J., Strippoli, R., et al. (2018). Caveolin-1 Modulates Mechanotransduction Responses to Substrate Stiffness through Actin-Dependent Control of YAP. *Cell Rep.* **25**, 1622-1635.e6.

Nasrin, S. R., Ganser, C., Nishikawa, S., Rashedul Kabir, A. M., Sada, K., Yamashita, T., Ikeguchi, M., Uchihashi, T., Hess, H. and Kakugo, A. (2021). Deformation of microtubules regulates translocation dynamics of kinesin. *Sci. Adv.* **7**,.

Price, A. J., Cost, A. L., Ungewiß, H., Waschke, J., Dunn, A. R. and Grashoff, C. (2018). Mechanical loading of desmosomes depends on the magnitude and orientation of external stress. *Nat. Commun.* **9**,.

Renn, J. P., Bhattacharyya, S., Bai, H., He, C., Li, H., Oberhauser, A. F., Marko, J. F., Makarov, D. E. and Matouschek, A. (2019). Mechanical unfolding of spectrin reveals a super-exponential dependence of unfolding rate on force. *Sci. Rep.* **9**, 11101.

Smith, M. L., Gourdon, D., Little, W. C., Kubow, K. E., Eguiluz, R. A., Luna-Morris, S. and Vogel, V. (2007). Force-induced unfolding of fibronectin in the extracellular matrix of living cells. *PLoS Biol.* **5**, 2243–2254.

Suman, S. K., Daday, C., Ferraro, T., Vuong-Brender, T., Tak, S., Quintin, S., Robin, F., Gräter, F. and Labouesse, M. (2019). The plakin domain of C. Elegans VAB-10/plectin acts as a hub in a mechanotransduction pathway to promote morphogenesis. *Dev.* **146**,.

Uttagomol, J., Ahmad, U. S., Rehman, A., Huang, Y., Laly, A. C., Kang, A., Soetaert, J., Chance, R., Teh, M. T., Connelly, J. T., et al. (2019). Evidence for the desmosomal cadherin desmoglein-3 in regulating YAP and Phospho-YAP in keratinocyte responses to mechanical forces. *Int. J. Mol. Sci.* **20**,.

Vahabikashi, A., Adam, S. A., Medalia, O. and Goldman, R. D. (2022). Nuclear lamins: Structure and function in mechanobiology. *APL Bioeng.* **6**,.

Yao, M., Qiu, W., Liu, R., Efremov, A. K., Cong, P., Seddiki, R., Payre, M., Lim, C. T., Ladoux, B., Mège, R.-M., et al. (2014). Force-dependent conformational switch of α-catenin controls vinculin binding. *Nat. Commun.* **5**,.

Zhong, C., Chrzanowska-Wodnicka, M., Brown, J., Shaub, A., Belkin, A. M. and Burridge, K. (1998). Rho-mediated contractility exposes a cryptic site in fibronectin and induces fibronectin matrix assembly. *J. Cell Biol.* **141**, 539–551.

Journal of Cell Science • Supplementary information
